# Young People’s Behavioral Intentions towards Low-Carbon Travel: Extending the Theory of Planned Behavior

**DOI:** 10.3390/ijerph18052327

**Published:** 2021-02-26

**Authors:** Xiaojian Hu, Nan Wu, Nuo Chen

**Affiliations:** 1 Jiangsu Key Laboratory of Urban ITS, Southeast University, Nanjing 210096, China; huxiaojian@seu.edu.cn (X.H.); 220183044@seu.edu.cn (N.C.); 2 Jiangsu Province Collaborative Innovation Center of Modern Urban Traffic Technologies, Southeast University, Nanjing 210096, China; 3 School of Transportation, Southeast University, Nanjing 210096, China

**Keywords:** low-carbon travel, young people, theory of planned behavior, structural equation modeling

## Abstract

With the rapid development of China’s economy and the rapid growth of the population, the demand for traffic is gradually changing from slow to fast, and the traffic congestion, air pollution, climate change and public health problems are becoming increasingly prominent. As residents’ willingness for low-carbon travel plays a crucial role in alleviating the problems caused by traffic, many studies pay attention to this aspect, but young people are still an obviously neglected group in the study of willingness for low-carbon travel. The novelty of this study lies in the extension of environmental concern and perceived moral obligation to the theory of planned behavior to explore the factors influencing young people’s low-carbon travel behavioral intention. The structural equation modeling is validated with a sample of 235 young respondents. The results show that attitude, perceived behavior control, environmental concern and perceived moral obligation have a significant positive correlation with young people’s low-carbon travel behavioral intention, while subjective norm has not. By revealing young people’s intention of low-carbon travel, this study could help to enhance the understanding of young people’s low-carbon travel choices, and could provide guidance for how to guide young people to choose low-carbon travel in the future.

## 1. Introduction

With the rapid development of the economy, urbanization and traffic motorization are developing rapidly, and the traffic demand is changing from slow to fast. Air pollution [1], climate change [2] and public health problems [3] caused by traffic are becoming increasingly prominent. The traffic sector consumes more than half of the world’s liquid fossil fuels each year and emits nearly a quarter of the energy-related carbon dioxide [4]. According to International Energy Agency, the global carbon dioxide emissions from traffic will grow at an annual rate of 1.7% by 2030, and at 3.4% in developing countries. Su et al. [5] pointed out that private cars are the main source of urban traffic energy consumption and carbon dioxide emissions, which means that travel with a private car is a high-carbon travel mode and will exacerbate the negative impact of traffic. In order to reduce carbon dioxide emissions from traffic, many traffic demand management/traffic policies have been developed around the world to encourage residents to choose low-carbon travel modes [6]. The 2011 China Energy Development Report defines low-carbon travel as a travel mode that can reduce carbon emissions during travel consciously adopted by travelers. Low-carbon travel advocates the formation of a “low-pollution, low-energy consumption” mode of traffic. To date, China has taken active measures to promote low-carbon travel mode, but has not achieved satisfactory results in low-carbon travel.

As the main body of traffic activities, residents’ travel behavior can affect the composition of traffic structure and is the most important factor that causes the adverse consequences of traffic. Hence, it is of great importance to guide residents to choose low-carbon travel modes. Numerous studies have investigated the behavior and intentions of individuals towards low-carbon travel in relation to different countries, regions, cultures and environmental conditions [7,8,9,10,11], however, these studies have been mainly aimed at ordinary residents, and have not tried to understand young people’s travel behavioral intention (BI). Among residents, young people are reported to have more pragmatic attitudes towards the ownership and usage of cars, and pay more attention to environmental and climate change issues [12,13]. Young people also appear to be particularly receptive to public transport, road charging, parking policies and other motor vehicle restriction arrangements [14]. In addition, young people are more likely to experience the impacts of climate change than older people, therefore, guiding young people to choose low-carbon travel modes is considered to be more efficient and convenient. While, to date, young people remain a remarkably neglected group in global traffic planning, cultivating the awareness of choosing low-carbon travel among young people needs to be put on the agenda.

Therefore, in order to guide young people to choose low-carbon travel modes, it is very meaningful to study young people’s travel behaviors. BI is widely believed to be the key to actual behavior [15]. Therefore, the primary objective of this study is to explore the critical factors influencing young people’s low-carbon travel BI. To achieve this objective, research hypotheses are first developed based on previous studies. Considering that young people might be more concerned about the environment and more responsible at the individual moral level, environmental concern (EC) and personal moral obligation (PMO) are added into the theory of planned behavior (TPB), which has been applied to behavioral assessment, including traveler intentions towards low-carbon travel options [16,17]. Then, structural equation modeling (SEM) is used to test out research hypotheses and to analyze how the factors affect the low-carbon travel BI of young people. Finally, the research results are compared and discussed with the previous studies to provide meaningful suggestions.

The rest of this study is organized as follows. Section 2 expounds the theoretical background and research framework, and gives the hypothesis of research variables. In Section 3, the research objects, questionnaires and data of this study are introduced, and the research methods are explained. Next is the results section, which analyzes the validity of the questionnaire to ensure the reliability of the results, and gives the results of the hypothesis of the variables in Section 2. Then, a discussion of the research results is provided and recommendations for low-carbon travel intentions are made. The last part is the conclusion, which gives the final summary for the research content of this study.

## 2. Literature Review and Hypothesis Development

The TPB proposed by Ajzen in 1985 has been widely used to investigate pro-environmental behavior (PEB). According to the TPB, people act after they have formed a BI, which in turn is mainly predicted by attitude (ATT) towards a specific behavior; subjective norm (SN), which represents the expectations of other important people; and perceived behavioral control (PBC), which is the sense of being able to perform the desired action [15,18]. The TPB emphasizes psychological factors of related behaviors and is a universal model that could predict and explain various PEBs, such as pollution reduction intention [19], choice of travel mode [10,20,21], energy-saving behavior [22,23,24], use of alternative transportation [25,26], low carbon consumption [27] and so on. Many meta-analytic reviews have confirmed that individual BI and actual behavior can be well explained and predicted by the TPB [28,29]. Choosing to use the TPB to predict the low-carbon travel BI of young people is considered appropriate.

Although TPB has received strong empirical support, it has been criticized for underestimating the impact of morality on environmental behavior [30]. Therefore, many previous studies have focused on extending the TPB model by introducing variables to improve the TPB’s explanatory capabilities. For example, Donald et al. [17] and Hyungsook [31] noted that individuals with a high degree of EC have a strong willingness to engage in PEB. They extended the TPB model by introducing EC and confirmed that EC has important influences on an individual’s PEB BI. Chen et al. [32] extended the TPB model by incorporating EC to predict consumers’ intention to visit green hotels. Albayrak et al. [33] pointed out that EC has proven to be one of the most popular variables used to study PEB. Zhang et al. [34], on the other hand, took EC as the respondents’ self-perceived responsibility for energy conservation. It is believed that people’s EC will affect people’s efforts to practice PEB [35] and many researchers studied and confirmed the positive relationship between EC and BI [32,33,36,37,38]. Moreover, Kaiser [39] proposed that when predicting consumers’ conservation BI, the model should contain a moral dimension, the explanatory power of the TPB in PEB BI could be improved by adding moral norms. Chan et al. [40] and López-Mosquera et al. [41] expanded descriptive norms based on the basic TPB to identify critical factors that influence the individual BI of consumers. Petschnig et al. [42] investigated the impact of individual norms on the intention to purchase alternative fuel vehicles. López-Mosquera et al. [41] argued that the inclusion of components of moral obligation can lead to a greater understanding of environmental intentions and behaviors. Chan and Bishop [40] studied the TPB extension of moral norms on recycling household waste in 2013 and Chen and Tung [32] studied the TPB extension of moral norms on visiting green hotels in 2014, and they all found that their results supported the moral norms extension of the TPB. In addition to the above extension factors, there are other factors that are added according to different research needs, for example, Wang et al. [43] and Lo et al. [9] combined energy knowledge and habit, respectively, with the TPB to investigate individual PEB BI. Zheng et al. [44] and Shi et al. [45] combined the technology acceptance model with the TPB to investigate young people’s willingness to rent a house and use urban-shared products, respectively. These additional variables expand and improve the effectiveness of the TPB in interpreting and predicting intention and behavior in given situations.

**Figure 1:**
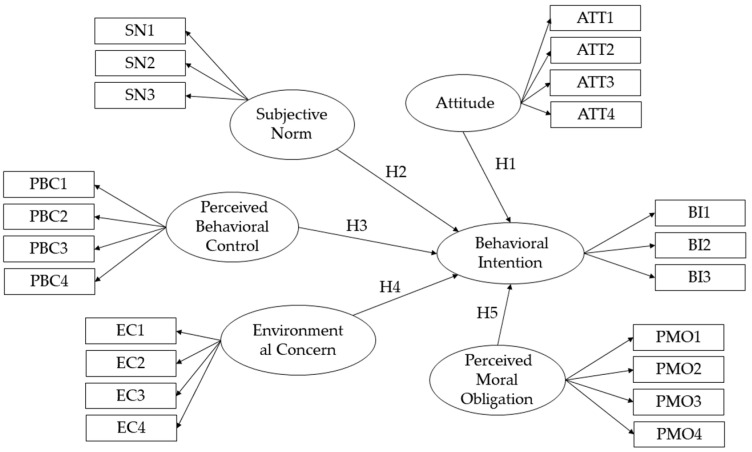
Expanded theory of planned behavior (E-TPB) model and survey items of E-TPB variables.

Therefore, on the basis of the original TPB and taking the group characteristics of young people and the research objective of this article into account, two variables, EC and PMO, are added to expand the original TPB in this study, and an expanded TPB (E-TPB) model with BI as the dependent variable is constructed to explore the influencing factors of young people’s low-carbon travel intention. Figure 1 displays the E-TPB model.

### 2.1. Attitude

As one of the three conceptually independent determinants of BI in the TPB, ATT refers to the degree to which an individual is positive or negative about a certain behavior [15,46,47].

Many studies have shown that individuals who are positive towards a certain behavior are more likely to have a strong intention to participate in this behavior [48] and ATT is among the most relevant predictors of PEB BI [41,43,46,49,50]. Hence, this study defines ATT as young people’s perceptions and tendencies of behavior towards low-carbon travel. If young people have a positive ATT towards low-carbon travel, then they will be more aware of the importance of low-carbon travel and are consequently more intent on engaging in low-carbon travel. Therefore, this study expects that ATT is positively related to low-carbon travel intentions and develops the following hypothesis:

H1. ATT is positively related to low-carbon travel BI.

### 2.2. Subjective Norm

SN refers to the social pressure that people feel to perform or not to perform one behavior [15]. SN also refers to an individual’s feelings of social pressure from another person or group [15,32,47].

Many previous studies applying the TPB have found that SN is a significant determinant of PEB [32,33,51], and have shown that the greater the external social pressure, the stronger the BI of individuals [23,29,52,53]. Hence, this study defines SN as the influence of external social pressure on the BI of young people to choose low-carbon travel modes. The greater the external social pressure, the stronger their BI. Thus, this study develops the following hypothesis:

H2. SN is positively related to low-carbon travel BI.

### 2.3. Perceived Behavior Control

PBC refers to an individual’s perception of the difficulty in performing a particular behavior [15]. Klöckner [47] said that PBC measures the degree to which an individual has the opportunity and ability to perform a behavior. Albayrak et al. [33] also thought PBC refers to whether an individual can easily consume a certain product or whether the consumption would be difficult.

In the field of PEB behavior, PBC has been studied and proved to be an important determinant of PEB BI [32,33,41]. Wang et al. [43] proved that PBC can importantly influence BI among residents in the context of energy-saving behavior in Beijing, China. The more effectively young people feel they can engage in the behavior, the more likely that they are willing to engage in the behavior. Thus, this study develops the following hypothesis:

H3. PBC is positively related to low-carbon travel BI.

### 2.4. Environmental Concern

Professors believe that people who care about the environment are more likely to engage in PEB. Albayrak et al. [33] found that EC has been proved to be one of the most prevalent variables in the study of PEB.

Previous studies have studied and confirmed the positive correlation between EC and BI [32,33,36,37]. Hence, this study expects that the more concerned young people are about the environment and the more aware they are of the constant impact of their actions on the environment, the more likely they will be willing to engage in PEB, which, in this study, means to choose low-carbon travel modes. Therefore, this study proposes the following hypothesis:

H4. EC is positively related to low-carbon travel BI.

### 2.5. Perceived Moral Obligation

PMO refers to the moral judgment of an individual on whether or not to act in a certain way [54]. Zhang et al. [55] thought PMO reflects to an individual’s self-expectation towards specific behaviors, which are mainly derived from the individual’s norms and values [55]. The influence of PMO on individual behavior mainly comes from internal pressure, such as responsibility and obligation. If their behavior is consistent with PMO, the individuals will feel proud, otherwise, they will feel guilty [56].

Previous studies have incorporated PMO in the TPB theoretical model [45,57], and results have shown that PMO can significantly increase the explanatory variance ratio of the original TPB model [23,57]. Ru et al. [19]and Wan et al. [58] have found that PMO has a significant positive impact on PEB BI. Theoretically, if young people consider that they have the responsibility and obligation to choose low-carbon travel modes, then their intention will be strengthened. Thus, this study proposes the following hypothesis:

H5. PMO is positively related to low-carbon travel BI.

## 3. Methodology

### 3.1. Research Objects

The objects of this research are young people aged from 18 to 40. According to the different research scope and depth, the age definition of the young people is also different. For example, in the Medium Term and Long Term Youth Development Plan (2016–2025) [59] issued by CPC (Communist Party of China) and SC (State Council), young people are defined as those aged from 14 to 35. In China, people aged 18 can start to get a driving license, and then they are able to travel more flexibly (whether low-carbon or not). Taking the objective of this study into account, the starting age of research objects is 18. Furthermore, the definition of young people should consider the appropriateness of the adolescence span, ensure the proportion of the population of young people in the total population is appropriate, and that the social status and main needs of the young people benefited by the policy are appropriate [59]. Therefore, in this study, young people are defined as those aged from 18 to 40. Due to age, young people experience environmental changes in a wider range and larger proportion in their lifetime than middle-aged and elderly people. As key stakeholders, young people must bear the consequences of past and present unfriendly environmental behaviors, which may cause them to be one of the powerful engines of PEB [60]. Compared with middle-aged and elderly people, young people seem to be more inclined to implement PEB and pay more attention to environmental and climate change issues [12,13], and are also particularly receptive to a series of ecologically friendly traffic management measures [14]. In addition, as the backbone force to inherit human civilization and promote social development, they are more responsible at the individual moral level. Therefore, for young people’s tendency towards PEB and the benefits their behaviors may bring, this study takes young people aged from 18 to 40 as the research object to study the influencing factors of their low-carbon travel intention.

### 3.2. Questionnaire Design

This study uses questionnaires to collect relevant data. The questionnaire of this study is designed to contain 7 main parts. The first part aims to collect demographic information of the research objects. The second part measures the factors that influence the research objects’ ATT towards low-carbon travel. The third and fourth parts, respectively, measure the SN and PBC of the research objects. The fifth and sixth parts, respectively, measure the EC and PMO of the research objects, which are the extension of the TPB in this study. The low-carbon travel BI is measured in Part 7.

This study selects mature scales and items from related studies to construct observation variables and to ensure the validity of the questionnaire. In other words, the items included in the questionnaire are referenced and improved based on previous research (listed in the last column of Table 1). Many studies in the past used a seven-point Likert scale as the questionnaire measurement method [42,51]. The seven-point Likert scale provides more choices and increases the possibility of meeting people’s objective reality. In addition, the sample BI displayed and described through the seven-point Likert scale is more accurate [42,61]. Therefore, this study uses a seven-point Likert scale in the questionnaire survey, from 1 for “strongly disagree” to 7 for “strongly agree”, to indicate the degree of agreement with each item. Table 1 shows the items used for data collection.

### 3.3. Data Collection

The data required for this study were collected from 1 October 2020 to 1 December 2020, and the questionnaire survey was completed online. Through the convenience sampling method, the questionnaires were randomly assigned to young people aged 18 to 40. Although the convenience sampling method may to some extent restrict the generalizability of the study, Yadav et al. [70] revealed that the results of random sampling among young people are effective and reliable. According to the sample selection method of Yadav et al. [70], the questionnaire was administered in groups through WeChat and QQ (China’s most popular social networks). Young people aged 18 to 40 received the survey invitation from their teachers, classmates, colleagues or friends in WeChat and QQ and filled out the questionnaire online. In this study, five hundred survey invitations were sent, and 301 online replies were obtained, for a recovery rate of 60.2%. After the data were cleaned, 235 completed valid questionnaires were obtained successfully.

### 3.4. Research Model

In order to examine the complicated interrelationships among the TPB’s latent variables of young people’s low-carbon travel BI, descriptive statistical analysis, reliability and structural validity are conducted using SPSS 25.0 to confirm that the data basically obey normal distribution, the data meet the structure assumption of SEM and the proposed model is applicable for further analysis. Then, SEM is estimated using Amos 26.0 to verify the relationship between variables [69].

## 4. Results

### 4.1. Descriptive Statistical Analysis

Among the respondents in this study, 57.45% (*n* = 135) are male and 42.55% (*n* = 100) are female. Of the respondents, 60.85% (*n* = 143) have a master’s degree or above. Most of the respondents are students (43.83%) and employees of enterprises (34.04%), and the monthly income of most of them is less than RMB 2000 (41.28%), which is related to the fact that most of them are students. The demographic characteristics of the respondents are shown in Table 2.

In order to understand the characteristics of data concentration and volatility, a descriptive statistical analysis is conducted on 235 valid questionnaires. Skewness and kurtosis are the normality test indexes of scale data. The maximum likelihood estimation method commonly used in SEM parameter estimation is not reliable in the correctness and stability of parameter estimation results when data deviate from the normal distribution. Therefore, the data analyzed by SEM must conform to the normal distribution. Under normal distribution, the skewness coefficient and kurtosis coefficient of the data are close to 0. The data does not fit the normal distribution when the absolute value of skewness > 3 or the absolute value of kurtosis > 8 [71,72]. In this study, both of above basically obey normal distribution and meet the data structure assumption of SEM (from Table 3).

### 4.2. Reliability and Validity

Reliability analysis refers to whether a group of items in a questionnaire measures the same concept, that is, how consistent are the questions internally. In general, the consistency of items is related to the measurement content, and the greater the value of Cronbach’s alpha, the greater the internal consistency. Previous studies have found that if Cronbach’s alpha coefficient is greater than 0.7, items are considered to have good consistency [65,67]. In this study, Cronbach’s alpha coefficients are all greater than 0.7 (from Table 4), so the consistency between items is good. Table 4 also shows the Cronbach’s alpha if an item is deleted, which indicates how much Cronbach’s alpha is likely to increase or decrease when an item is deleted. As shown in the “Cronbach’s Alpha if Item Deleted” column, when ATT3, SN2, PBC2, EC2, PMO2 or BI2 is deleted, Cronbach’s alpha is likely to decrease more. Corrected item–total correlation (CITC) is used to determine whether an item needs to be deleted or not. The CITC column refers to the Pearson correlation coefficient between each particular item and other items. In this study, all values in the CITC column are greater than 0.3, so there is no need to delete items. Thus, the reliability of this study is verified.

To ensure the structural validity of the questionnaire, the commonly used exploratory factor analysis (EFA) is used to test the structural validity of the questionnaire. In this study, BI is taken as the result variable, three items reflecting BI are analyzed separately by EFA (From Table 5) and then the remaining items are analyzed by EFA (from Table 6). According to the initial EFA findings shown in Table 5 and Table 6, the constructs’ factor loadings are scaled from 0.519 to 0.957, higher than the recommended threshold of 0.50 [57,67]. The rate of variance of extraction sums of squared loadings are all higher than the recommended threshold of 10%. Additionally, the KMO (Kaiser–Meyer–Olkin) and Bartlett’s test are used as the criteria for the test of correlation and independence between variables, and the structural validity test results shown in Table 5 and Table 6 mean that the questionnaires of this study are suitable for further analysis (when KMO > 0.7 and Bartlett *p* ≤ 0.01, the questionnaires are suitable) [72]. The proposed model shows the necessary validity and reliability and is ready for further analysis.

### 4.3. Hypotheses Analysis

The SEM is constructed using Amos 26.0 in this study. Firstly, the model fitting results of structural analysis cannot meet the suggested threshold. Through numerous experiments on SEM, Charles [73] once noted that most models cannot meet the fitting criteria the first time due to data deviation or problems with the models themselves. Hence the modification indices are applied and, after modification, a good model fitting is obtained (χ^2^/df = 2.343 (the ratio of chi-square to the degree of freedom), NFI = 0.925(normal of fit index), CFI = 0.955 (comparative fit index), RMSEA = 0.076 (root mean square error of approximation)) [57,67]. Table 7 shows the results of model fit indices before and after model revision.

**Figure 2:**
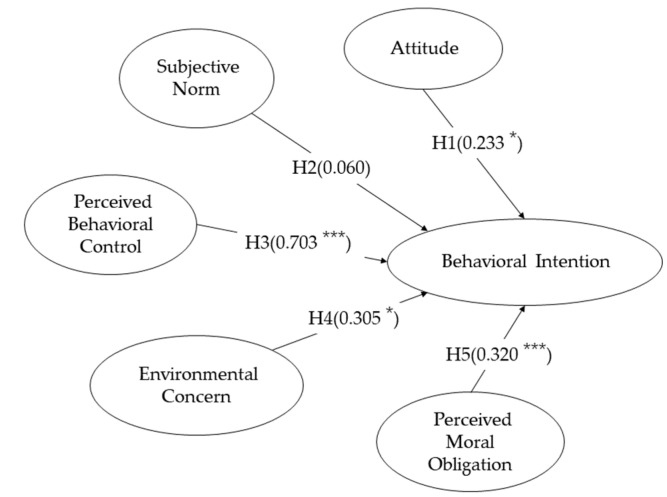
Hypothesis testing results of E-TPB model.

The results of the hypothesis test are shown in Figure 2 and Table 8. As shown in Table 8, the standardized path coefficients of ATT, PBC, EC and PMO are within the two-tailed 95% confidence interval, meaning that ATT, PBC, EC and PMO all have a positive and significant effect on BI of low-carbon travel (b_H1_ = 0.233, *p* < 0.05; b_H3_ = 0.703, *p* < 0.001; b_H4_ = 0.305, *p* < 0.05; b_H5_ = 0.320, *p* < 0.001), therefore, H1, H3, H4 and H5 are supported. However, SN does not have a positive and significant effect on BI of low-carbon travel, so H2 is rejected.

## 5. Discussion

This study designs an E-TPB model to examine influencing factors of young people’s low-carbon travel BI.

The research results of this study show that young people’s ATT towards low-carbon travel are positively correlated with their BI towards low-carbon travel, which means that young people who have favorable attitudes towards low-carbon travel would generally intend to choose low-carbon travel in their daily lives. This finding is consistent with previous studies by López-Mosquera et al. [41] and Greaves et al. [49]. However, this study does not find a significant positive correlation between the SN of low-carbon travel and BI of low-carbon travel, and this relationship was not confirmed in the study of López-Mosquera et al. [41]. This means that young people are not easily influenced by the opinions of those around them when considering low-carbon travel. Arvola et al. [74] once argued that SN may not be fit for use with the TPB because SN is generally group-based and, to some extent, it may not reflect an individual’s own norms towards a particular behavior. Furthermore, the respondents in this study are young people, they have active thinking, have independent lives and have a strong sense of independence, so when making decisions, they will not be easily influenced by the opinions of people around them. However, this finding is in contradiction with the findings of Ha and Janda [46], who found that there is a significant positive correlation between Korean consumers’ BI of purchasing energy-efficient home appliances and SN. Cultural differences between Korea and China may well have contributed to this, as once explained by Olsen et al. [50] that cultural differences can cause different levels of social pressure towards the behavior. Since the birth of the TPB, many researchers have confirmed that PBC is an important determinant of supporting the TPB BI [32,63]. The research results of this study also confirm the importance of PBC in predicting young people’s low-carbon travel BI. This result means that when young people have more control over the conditions and ability to choose low-carbon travel, they are more likely to do so.

For the additional variables, this study shows that EC has a significant positive impact on young people’s low-carbon travel BI, which is the same as the research results of Shi et al. [45]. This shows that young people’s concern about the environment can make them more inclined to choose low-carbon travel. From the young people’s point of view, low-carbon travel seems to contribute a lot to improving the environment. As a result, they tend to engage in environmentally friendly travel behaviors. Additionally, that the PMO of young people significantly positively influences their low-carbon travel BI is confirmed in this study. It shows that young people’s subjective judgment on the correctness of low-carbon travel is the main factor affecting young people’s low-carbon travel BI. The conclusion of Ru et al. [19] on the intention of young people to reduce PM2.5 (Particulate Matter 2.5) also confirms the important influence of PMO. When young people have a strong sense of responsibility and obligation towards low-carbon travel, their willingness to choose low-carbon travel will increase.

From the practical point of view, this study provides the influence of ATT, SN, PBC, EC and PMO on young people’s low-carbon travel BI. The results of this study show that ATT, PBC, EC and PMO have a significant positive correlation with young people’s low-carbon travel BI while SN does not. Among all the factors affecting young people’s low-carbon travel BI, PBC is the determining factor (the main predictor) for young people to consider low-carbon travel. When young people think about low-carbon travel, compared with other variables, PBC can better predict the low-carbon travel intention of young people. This is because contemporary young people have a strong ability for independent thinking, and they expect to have a high ability of control over their behaviors. On the other hand, this also confirms why SN has no significant correlation with young people’s low-carbon travel BI, that is, young people will not be easily influenced by the opinions of people around them when making decisions. Given the above findings, interventions to incentivize young people to choose low-carbon travel should target the PBC first, since PBC can significantly correlate with BI. Measures should be implemented to enable young people to feel that they could have a high ability of control over choosing low-carbon travel. For example, time accuracy of low-carbon travel can be improved to ensure that young people can arrange their time well when choosing low-carbon travel; comprehensive and convenient low-carbon travel facilities should be arranged by relevant authorities to make sure that low carbon travel is convenient. This study also shows that PMO can also affect young people’s intention of low-carbon travel to a large extent. Young people’s willingness for low-carbon travel is morally right, and PMO reflects an individual’s self-expectation towards specific behaviors, which are derived from the individual’s norms and values. PMO can provide internal satisfaction, which in turn can encourage BI. Targeting the PMO of young people may offer opportunities to change their behavior. In order to improve the PMO towards young people’s low-carbon travel, society may promote the publicity of low-carbon travel and make young people realize that choosing low-carbon travel is their responsibility to society. In sum, the results of this study demonstrate that the E-TPB can be used as a conceptual framework to understand the influencing factors of young people’s low-carbon travel BI.

## 6. Conclusions

Understanding influencing factors of young people’s low-carbon travel BI is of great theoretical and practical significance for guiding young people to actively participate in low-carbon travel. This study mainly studies the relationship between ATT, SN, PBC, EC, PMO and BI of young people’s low-carbon travel, and discusses the key factors affecting the BI of young people’s low-carbon travel. By extending the TPB, this study builds and tests an E-TPB model to explain young people’s low-carbon travel BI, the collected data are analyzed, the research hypotheses are tested and, finally, four of the five hypotheses are supported in this study. The results show that ATT, PBC, EC and PMO have a significant positive correlation with young people’s low-carbon travel BI. Contrary to some previous studies, SN has no significant correlation with young people’s low-carbon travel BI, which may be related to the research objects in this study.

This study attempts to explore young people’s low-carbon travel BI, the results of which can help to enhance the understanding of young people’s low-carbon travel choices, and can provide guidance for how to guide young people to choose low-carbon travel in the future. Young people will become the backbone of society, and guiding the travel choices of young people reasonably will change the future of the traffic structure. If young people can be reasonably guided to practice PEB and to choose low-carbon travel modes, and the travel choices of young people can form an organic resonance with the goal of ecological civilization construction, this will help to alleviate and eliminate the urgent ecological and environmental problems caused by traffic, such as excessive energy consumption, environmental damage and the decline of ecological carrying capacity.

In addition, this study also has some limitations. First is the limitation of online sample surveys. Due to the existence of some potential problems in the online survey used in this article, the influencing factors of young people’s low-carbon travel BI and relevant suggestions given in this study may not be universally applicable. Second is the limitation of hypothesis relations. Studying the relationship between the ATT, SN, PBC, EC, PMO and BI of young people’s low-carbon travel may be not enough, as it is also necessary to determine whether the independent variables influence each other. In future research, if the research samples can be expanded, the online survey can be perfected and combined with offline surveys and more complex research frameworks can be constructed to examine meaningful correlations/interactions, then more universal and reasonable research conclusions can be obtained to a great extent.

## Figures and Tables

**Table 1:** Survey items of young people’s low-carbon travel BI.

Latent Variable		Measurement Item	Sources
ATT	ATT1	When I travel, I will consider whether the way to travel is low-carbon.	[22,46,60]
	ATT2	I think low-carbon travel can solve the problem of environmental pollution, which is better than other ways of travel.	
	ATT3	I think low-carbon travel is meaningful.	
	ATT4	I have a favorable attitude toward low-carbon travel.	
SN	SN1	The people around me are used to low-carbon travel.	[32,45,62]
	SN2	People around me expect me to choose low-carbon travel.	
	SN3	The promotion of low-carbon travel by people around me has a great influence on the way I choose to travel.	
PBC	PBC1	I believe I will adopt low-carbon travel even if it takes more time than other travel.	[63,64,65]
	PBC2	I believe I will adopt low-carbon travel even if others say it is not that important.	
	PBC3	I believe that as long as I choose low-carbon travel, it will definitely have some positive impact on the environment.	
	PBC4	I can choose low-carbon travel as long as I want to.	
EC	EC1	I think environmental issues are related to human survival.	[35,65,66]
	EC2	I think people should protect the environment.	
	EC3	I think people must live in harmony with nature.	
	EC4	I’m very concerned about the environment.	
PMO	PMO1	Choosing low-carbon travel conforms to my environmental protection principle.	[67,68,69]
	PMO2	I think It is my obligation to choose low-carbon travel in my daily life.	
	PMO3	I think it is my responsibility to choose low-carbon travel in my daily life.	
	PMO4	I would feel guilty if I did not choose low-carbon travel in my daily life.	
BI	BI1	I intend to participate in low-carbon travel immediately.	[11,45]
	BI2	I will strive to participate in low-carbon travel in the near future.	
	BI3	I am willing to participate in low-carbon travel.	

Note: ATT means attitude, SN means subjective norm, PBC means perceived behavioral control, EC means environmental concern, PMO means personal moral obligation and BI means behavioral intention.

**Table 2:** Demographic characteristics of the respondents.

Feature	Type	Frequency	Percentage/%
Gender	Male	135	57.45
	Female	100	42.55
Education	Senior high school or below	4	1.70
	College or bachelor’s degree	88	37.45
	Master’s degree or above	143	60.85
Profession	Student	103	43.83
	Employees of government agencies or public institutions	32	13.62
	Employees of enterprises	80	34.04
	Individual practitioner	8	3.40
	Others	12	5.11
Income	<RMB 2000	97	41.28
	RMB 2001–5000	24	10.21
	RMB 5001–8000	36	15.32
	RMB 8001–10,000	24	10.21
	>RMB 10,001	54	22.98

**Table 3:** Skewness and kurtosis.

Latent Variable	Measurement Item	Mean	Skewness		Kurtosis	
			Skewness	Std. Error of Skewness	Kurtosis	Std. Error of Kurtosis
ATT	ATT1	4.69	−0.309	0.159	−0.800	0.316
	ATT2	5.80	−1.083	0.159	0.998	0.316
	ATT3	5.50	0.374	0.159	−0.522	0.316
	ATT4	6.23	−1.406	0.159	3.969	0.316
SN	SN1	4.59	0.147	0.159	−0.698	0.316
	SN2	4.54	−0.013	0.159	−0.717	0.316
	SN3	4.65	0.248	0.159	−0.610	0.316
PBC	PBC1	4.20	−0.139	0.159	−0.728	0.316
	PBC2	4.86	−0.368	0.159	−0.677	0.316
	PBC3	5.45	−0.607	0.159	0.279	0.316
	PBC4	5.70	−0.693	0.159	0.323	0.316
EC	EC1	6.10	−2.105	0.159	5.837	0.316
	EC2	6.49	−1.385	0.159	0.870	0.316
	EC3	6.51	−1.978	0.159	3.618	0.316
	EC4	5.63	−0.668	0.159	0.129	0.316
PMO	PMO1	5.74	−1.028	0.159	1.053	0.316
	PMO2	5.33	−0.976	0.159	0.854	0.316
	PMO3	5.50	−1.030	0.159	1.244	0.316
	PMO4	4.01	−0.024	0.159	−1.089	0.316
BI	BI1	5.00	−0.473	0.159	−0.159	0.316
	BI2	5.47	−0.966	0.159	1.116	0.316
	BI3	5.67	−0.924	0.159	1.026	0.316

**Table 4:** Reliability test results.

Latent Variable	Measurement Item	Cronbach’s Alpha	Cronbach’s Alpha if Item Deleted	CITC
ATT	ATT1	0.727	0.744	0.380
	ATT2		0.582	0.562
	ATT3		0.526	0.651
	ATT4		0.672	0.437
SN	SN1	0.864	0.775	0.779
	SN2		0.762	0.790
	SN3		0.882	0.660
PBC	PBC1	0.807	0.766	0.610
	PBC2		0.664	0.778
	PBC3		0.784	0.547
	PBC4		0.776	0.575
EC	EC1	0.818	0.746	0.625
	EC2		0.735	0.679
	EC3		0.742	0.635
	EC4		0.772	0.580
PMO	PMO1	0.887	0.854	0.672
	PMO2		0.786	0.842
	PMO3		0.812	0.797
	PMO4		0.884	0.676
BI	BI1	0.925	0.929	0.800
	BI2		0.854	0.895
	BI3		0.890	0.841

Note: **CITC** means corrected item–total correlation.

**Table 5:** Structural validity test results of BI.

Latent Variable	Measurement Item	Factor Loading
BI	BI1	0.908
	BI2	0.957
	BI3	0.933
Extraction Sums of Squared Loadings	Total	2.610
	% of Variance	86.994
	Cumulative%	86.994
Kaiser–Meyer–Olkin Measure of Sampling Adequacy		0.728
Bartlett’s Test of Sphericity	Approx. Chi-Square	572.843
	Sig.	0.000

**Table 6:** Structural validity test results of ATT, SN, PBC, EC and PMO.

Latent Variable	Measurement Item	Factor Loading				
		1	2	3	4	5
ATT	ATT1	0.750				
	ATT2	0.697				
	ATT3	0.520				
	ATT4	0.777				
SN	SN1		0.799			
	SN2		0.858			
	SN3		0.768			
PBC	PBC1			0.746		
	PBC2			0.734		
	PBC3			0.519		
	PBC4			0.767		
EC	EC1				0.799	
	EC2				0.900	
	EC3				0.907	
	EC4				0.753	
PMO	PMO1					0.589
	PMO2					0.682
	PMO3					0.547
	PMO4					0.798
Extraction Sums of Squared Loadings	Total	5.688	2.429	2.126	2.049	2.037
	% of Variance	29.934	12.786	11.189	10.786	10.723
	Cumulative%	29.934	42.721	53.909	64.695	75.418
Kaiser–Meyer–Olkin Measure of Sampling Adequacy		0.897				
Bartlett’s Test of Sphericity	Approx. Chi-Square	3326..230				
	Sig.	0.000				

**Table 7:** Measurement of model fit indices.

Fit Indices	Criteria	Before Model Correction	Model Adaptation Judgment	After Model Revision	Model Adaptation Judgment
χ2/df	<3	3.667	No	2.343	Yes
NFI	>0.9	0.778	No	0.925	Yes
CFI	>0.9	0.825	No	0.955	Yes
RMSEA	<0.08	0.148	No	0.076	Yes

Note: χ^2^/df means the ratio of chi-square to the degree of freedom, NFI means normal of fit index, CFI means comparative fit index and RMSEA means root mean square error of approximation.

**Table 8:** Results of the hypothesis test.

Hypotheses	Path Correlation	Coefficient	SE	*t*-Value	*p*	Results
H1	ATT->BI	0.233	0.066	2.919	0.037 *	Supported
H2	SN->BI	0.060	0.071	0.931	0.220	Not Supported
H3	PBC->BI	0.703	0.069	8.323	***	Supported
H4	EC->BI	0.305	0.078	3.726	0.03 *	Supported
H5	PMO->BI	0.320	0.059	3.924	***	Supported

Note: * means *p* < 0.05, ** means *p* < 0.01, and *** means *p* < 0.001.

## Data Availability

The data presented in this study are available on request from the corresponding author. The data are not publicly available because the data in this article were obtained through a questionnaire survey, this study was supported by the respondents, but at the same time, the authors promised that respondents’ information would not be made public.
